# Supplementary material for: Non-invasive prenatal paternity testing by analysis of Y-chromosome mini-STR haplotype using next-generation sequencing
Source: PLoS One. 2022 Apr 1;17(4):e0266332. doi: 10.1371/journal.pone.0266332 (PMC8974964; doi:10.1371/journal.pone.0266332)
Supplement: S1 Table — (DOCX) [file pone.0266332.s001.docx]

**S1 Table. Primers and repeat motif of 12 Y-chromosome mini-STR loci without barcoding sequences**

| **Locus** | **Primer** | **Sequence (5＇ to 3＇)** | **Repeat motif** |
| --- | --- | --- | --- |
| DYS439 | Forward | tagatgataaataacagatagatgatGaat | [GATA]n |
|  | Reverse | GAtctatctatctatcAGAAT |  |
| DYS437 | Forward | ttcactgcaagccatgcctggttGaactac | [TCTA]n[TCTG]2[TCTA]4 |
|  | Reverse | tagatagacatcattcaTagatgatag |  |
| DYS643 | Forward | gccatgcctgAttaaactactg | [CTTTT]n |
|  | Reverse | gtaaaCagttttaaCaagaaa |  |
| DYS393 | Forward | taCtctatcttgaattaatagattcaa | [AGAT]n |
|  | Reverse | tagagcttctataccttctaagAtgtatgtc |  |
| DYS570 | Forward | tagaaatcctggctgtAtcctccaagttcc | [TTTC]n |
|  | Reverse | tctcttgtttacctatAcatcacctatct |  |
| DYS392 | Forward | ttaaacctaccaatccTattccttagt | [TAT]n |
|  | Reverse | gtttgttatttaaTagccaagaaggaaTac |  |
| DYS549 | Forward | tagTtaggtaaagagCaagatgatagat | [GATA]n |
|  | Reverse | ttccatttgtgattAtgtttatgtagat |  |
| DYS460 | Forward | tctgcctatcatttattatgtatCtgtct | [ATAG]n |
|  | Reverse | aataccagaTgaatctgacacctctg |  |
| DYS458 | Forward | tcttCtatttgatTgcattacaatagat | [GAAA]n |
|  | Reverse | aagcatgagcTaccacgTccac |  |
| DYS576 | Forward | caacatagcaTgacctcatctctga | [AAAG]n |
|  | Reverse | agAagatgCTagtaataagcAtattt |  |
| DYS438 | Forward | aacagtatacagattgtatgaCaagt | [TTTTC]n |
|  | Reverse | gTcaacaagagtgaaTctccattAcaT |  |
| DYS533 | Forward | tacTtatcatcttGctagctagctat | [ATCT]n |
|  | Reverse | tcaTccaaacaatagaaCatgata |  |
