# Supplementary material for: Non-invasive prenatal paternity testing by analysis of Y-chromosome mini-STR haplotype using next-generation sequencing
Source: PLoS One. 2022 Apr 1;17(4):e0266332. doi: 10.1371/journal.pone.0266332 (PMC8974964; doi:10.1371/journal.pone.0266332)
Supplement: S2 Table — (DOCX) [file pone.0266332.s002.docx]

**S2 Table. Quantities of the 24 cffDNA extracts**

| Case No. | CffDNA concentration (pg/µL) | Case No. | CffDNA concentration (pg/µL) |
| --- | --- | --- | --- |
| 1 | 41.1 | 13 | 62.8 |
| 2 | 48.4 | 14 | 39.7 |
| 3 | 59.8 | 15 | 50.4 |
| 4 | 72.8 | 16 | 63.7 |
| 5 | 83.3 | 17 | 57.2 |
| 6 | 47.4 | 18 | 88.2 |
| 7 | 60.5 | 19 | 36.6 |
| 8 | 77.3 | 20 | 49.7 |
| 9 | 43.2 | 21 | 38.5 |
| 10 | 46.8 | 22 | 76.5 |
| 11 | 69.9 | 23 | 98.1 |
| 12 | 58.2 | 24 | 64.2 |
