# Supplementary material for: Non-invasive prenatal paternity testing by analysis of Y-chromosome mini-STR haplotype using next-generation sequencing
Source: PLoS One. 2022 Apr 1;17(4):e0266332. doi: 10.1371/journal.pone.0266332 (PMC8974964; doi:10.1371/journal.pone.0266332)
Supplement: Attachment — Submitted filename: Response to Reviewers.docx [file pone.0266332.s005.docx]

Dear Dr. Chan,

Thank you for giving us another chance to revise our manuscript entitled “Non-invasive prenatal paternity testing by analysis of Y-chromosome mini-STR haplotype using next-generation sequencing”. We appreciate the constructive criticism from the reviewers, and have made the necessary changes in the rebuttal letter and manuscript. The revised portion in the manuscript is marked in yellow. The responses to the reviewers’ comments are as follows.

**Reviewer #2:** Dear colleagues:
In this current version, I believe that Song et al. have addressed most of the criticisms raised by the reviewers in the first submission. However, I'm still VERY CONCERNED about the fact that the authors are proposing a paternity test based exclusivelly on Y-STR haplotypes. In fact, my major comments, which I copy again below, were not responded by the authors.

I would like to stress that unlike autosomal STR genotypes, IDENTICAL Y-STR haplotypes are found in the population because the Y-chromosome is passed on from father to son without recombination. Thus, I strongly suggest that the authors at least acknowledge in the manuscript the possibility of false-positive results due to the inheriteance mode of Y-STR haplotypes. And ideally, the authors could also recommend that the Y-STR results must be confirmed with autosomal STR markers when a sample can be safely collected from the fetus/baby (e.g. after birth or miscarriage).

FROM PREVIOUS REVIEW:
First, even though a CPI is calculated to show some degree of uncertainty to the paternity tests performed, we should keep in mind that the Y-STR haplotype represents only one locus in the genome. Thus, this Y-STR paternity analysis would be similar to accepting the use of only one autosomal locus as sufficient to determine paternity with confidence (which can also show greater than 99% CPIs depending on the locus, frequencies and alleles scored).

Second, unlike the autosomal loci, the Y-chromosome is inherited from father to son without recombination. This mode of inheritance creates the potential for several men in the population to share the exact same Y-STR profile (i.e. they belong to the same lineage). For instance, if the paternal grandfather of one of those fetuses had four brothers and each of those brothers (including the grandfather himself) had four sons, and each of those 16 men had two sons, we would have, in this family alone, 52 individuals that would, in theory, match the fetus’ Y-STR profile. This isn’t the case when analyzing autosomal STR profiles (with at least a dozen loci each) due to recombination. In other words, only identical twins are expected to share the same STR profile in the population while several men can (and do) share the same Y-STR haplotype, creating the real potential of false positive results.

For these reasons, unlike it’s implied in the title and throughout manuscript, Y-STR haplotype results alone should not be used to confirm paternity. Instead, it could be used to “exclude” or “not exclude” a male from being a potential father of a fetus. Especially in the forensic setting, where paternity tests can have broad impacts on people's lives, conclusions should always be confirmed with more reliable methods such as genotyping multiple autosomal STR loci.

Answer: We apologize for not responding your major comments last time. “Unlike the autosomal loci … autosomal STR genotyping to determine paternity.” has been added in the manuscript.

**Reviewer #3:** All required clarifications and explanations in the text have been made. The description of the figures as 'redundant' was meant to signify that they served no purpose and could be excluded - but their retention is not a problem. I understand the underlying NGS data will be made available in due course, even thought the authors cannot meet this obligation at the moment.

Answer: Thanks for the suggestion. If possible, I hope the description of the figures can be retained. In addition, NGS data has been uploaded to GEO, and the relevant accession number is GSE186434.
